# Medicine and Pharmaceutical Research

**Published:** 1904-09-10

**Authors:** 


### MEDICINE AND PHARMACEUTICAL RESEARCH.

The article by Messrs. Wippell Gadd and Sydney C. Gadd, of which we publish an abstract in another column, is one which contains a very considerable degree of interest for members of the medical profession. In its more technical details it is concerned with practical pharmaceutical processes which in their more immediate relationship appeal to the expert pharmacist. But even those, in principle at least, are not destitute of claim upon the attention of the practitioner of medicine. The medical curriculum of recent years—perhaps necessarily so—has become burdened with many subjects that are hardly of ready application to the daily routine of professional life, and one of the results of this development has been a decline in the education of the student in practical materia medica and pharmacy. The old apprenticeship system, whatever its faults, had, at least, this advantage—that the pupil gained a practical knowledge of the various materials and preparations used as medicines. This, quite inexcusably as we think, has not been fully borne in mind in the modern curriculum, and as a consequence the art of prescribing has sensibly declined. It would be greatly to the benefit of practical medicine were members of the profession more generally competent to appreciate the work of the pharmacist and to co-operate with him for a common purpose. In the paper above referred to we have an illustration of the advances and improvements which may be effected by original pharmaceutical research. By a series of experiments the writers show how the official process of making the liquid extract of nux vomica can be modified so as to obtain a more satisfactory preparation. And there can be no difficulty in following the principles of these experiments or the conclusion to which they point. In spite of the unceasing production of new remedies, many of which enjoy but a brief day, it still remains true that the great majority of the agents on which the physician must rely in the treatment of disease have long been within the knowledge of the profession. What are wanted are reliable and convenient forms in which these traditional medicines can be employed. That great steps have been taken towards this end is certain. But there is no finality about such a process. The efforts of pharmacists must be continued and enlarged. What we should like to see is a keener and more intelligent interest in these efforts on the part of the medical profession. This would be well for the profession, and it would be well also for the art of pharmacy. There are numerous opportunities for improvement even in the official processes of the last edition of the British Pharmacopœia. Work which shows this to be the case, and indicates the line of improvement, is not a mere matter of academic pharmacy. It has the closest possible concern with successful therapeutics. There can be no doubt that were the physician more definitely to formulate his demands, a more precise application of chemistry and pharmacy would go far to meet these demands. And to follow the steps by which this end should be attained would not only be an education to the physician, but would equip him with a confidence in the materials of his art which can never be held towards substances that boast a mysterious origin and possess often a mere fictitious importance. It is upon this common basis that medicine and pharmacy meet; and the more completely the physician is in a position to appreciate the actual and potential value of the pharmacist as the source from which the art of therapeutics is to derive efficient and convenient remedial agents, the more fruitful this meeting-ground is likely to prove. The claims upon the medical practitioner are indeed numerous and varied. Some of them have a fascination and a wide interest to which the art of treatment can hardly pretend. Yet it must be remembered that this art is the first duty and proudest responsibility of the physician, and nothing which concerns it can be alien to his interest. Thus, at least so it seems to us, his attention may occasionally be directed to a piece of practical pharmaceutical research, first as important in itself, and secondly as illustrating services and achievements which bear directly upon the most urgent aspect of medical responsibility.

